# No-Reflow During Coronary Interventions: A Narrative Review

**DOI:** 10.3390/jcm14227976

**Published:** 2025-11-11

**Authors:** Sara Malakouti, Ahmed Hashim, Marco Frazzetto, Bernardo Cortese

**Affiliations:** 1 DCB Academy, 20136 Milano, Italy; dr.saramalakouti@gmail.com (S.M.); ahmed.hashim.fl@gmail.com (A.H.); marcofrazzetto7@gmail.com (M.F.); 2 Cardiovascular Research Group, Fondazione Ricerca e Innovazione Cardiovascolare, 20143 Milano, Italy; 3 Cardiovascular Medicine Department, Faculty of Medicine, Ain Shams University, Cairo 1181, Egypt; 4 Harrington Heart & Vascular Institute, University Hospitals Cleveland Medical Center, Cleveland, OH 44106, USA

**Keywords:** no-reflow phenomenon, PCI, microvascular obstruction, myocardial reperfusion, coronary interventions

## Abstract

The coronary no-reflow phenomenon remains a daunting and unresolved barrier during percutaneous coronary procedures, especially for acute coronary syndrome. Despite successful epicardial artery patency restoration, decreased microvascular perfusion leads to unfavorable outcomes such as ventricular remodeling, progression of heart failure, and increased mortality. This review provides a new, integrative informative perspective by combining multifactorial pathophysiology, which includes systemic inflammation, thrombogenicity, ischemia–reperfusion injury, and distal embolization, with advances in diagnostic imaging, such as cardiac magnetic resonance and computed tomography. Therapeutic options, including antithrombotic regimes, vasodilators, and mechanical adjuncts, are evaluated in the context of developing debates and unmet clinical needs. Importantly, we provide feasible future directions for artificial intelligence-based predictive modeling and targeted microvascular treatments. This comprehensive review fills a significant gap, aiming to inform personalized approaches and improve both short- and long-term outcomes in this high-risk patient population.

## 1. Clinical Implications

1. No-reflow phenomenon represents a complex, multifactorial challenge that profoundly compromises myocardial reperfusion and independently predicts adverse clinical outcomes post-coronary intervention in ACS patients.
2. Although biomarker identification and imaging modalities have improved early diagnosis and risk stratification, effective, standardized preventive therapies are still elusive.
3. Current pharmacological and mechanical management strategies yield inconsistent results, emphasizing the critical need for personalized, mechanism-driven treatment paradigms.
4. Emerging technologies, including artificial intelligence-driven risk prediction and novel microvascular-targeted therapies, offer promising avenues for precision medicine to mitigate no-reflow and improve patient prognosis.

## 2. Introduction

Acute myocardial infarction is one of the major causes of mortality in both developed and developing countries. The advancement of percutaneous coronary intervention over the last decade has paved the way for safer and efficient interventions [1]. However, the restoration of the flow in the infarct-related artery may not be associated with full restoration of the microvascular circulation, whose angiographic pattern is named the coronary slow flow or no-reflow (CNR) phenomenon. CNR is defined as failure to achieve proper tissue vascularization despite relieving the occlusion in the target vessels [2]. The CNR phenomenon occurs at variable frequencies across the spectrum of the acute coronary syndrome (ACS), with the highest rate in patients with ST elevation myocardial infarction [3,4]. Multiple procedural and patient-related risk factors have been associated with CNR, including plasma glucose levels, diabetes mellitus, and aspiration thrombectomy [5]. In the following review, we are going to go through different pathophysiological and technical challenges related to the development, prediction, and management of CNR.

## 3. Methodology

**Figure 1:**
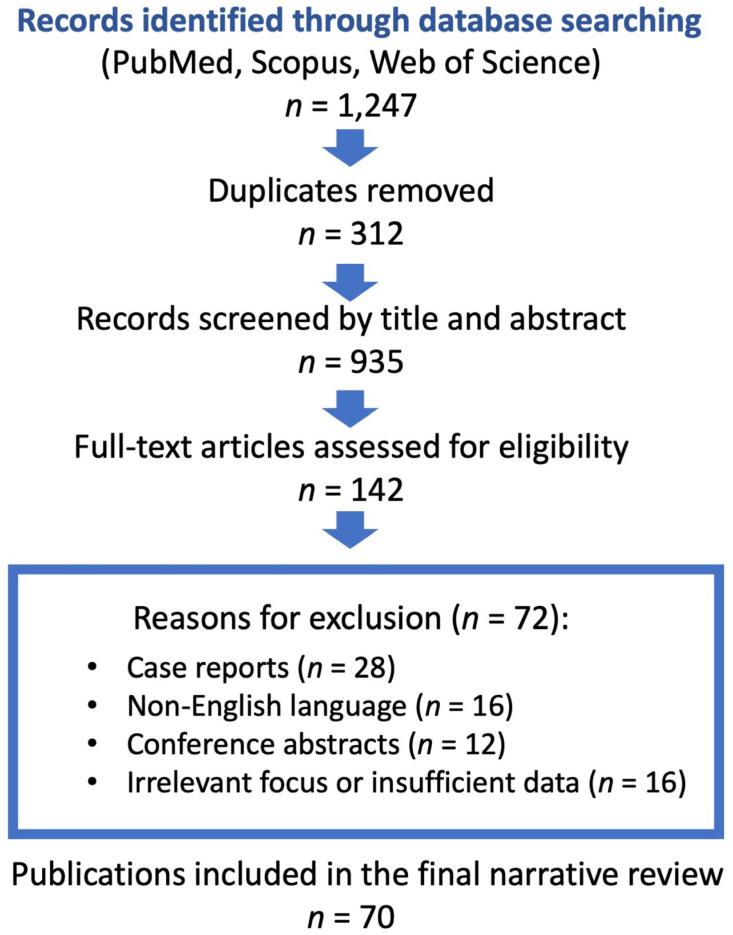
Flow diagram of the literature selection process.

A thorough search of the literature was conducted on the PubMed, Scopus, and Web of Science databases to identify peer-reviewed publications dated between January 2000 and September 2025. The search terms were “no-reflow phenomenon,” “microvascular obstruction,” “percutaneous coronary intervention,” and “acute coronary syndrome.” The initial search yielded 1247 records, before removing 312 duplicative records. Therefore, in total, 935 unique records were screened after removing duplicates (934 records screened using title and abstract), which led to 142 articles being screened in the full text. Using the inclusion and exclusion criteria, 70 articles were included in this narrative review (Figure 1). Articles were included in the review if they contained mechanisms, imaging, and/or management of the no-reflow phenomenon following PCI. Exclusion criteria were case reports, articles not in English, and conference abstracts.

### 3.1. Prevalence and Epidemiology

The development of CNR varies widely across the spectrum of acute coronary syndrome patients. The incidence of CNR in STEMI patients varies across different centers and geographical areas [6,7,8]. In a cohort of STEMI patients undergoing primary PCI, the nomogram risk model reported an 18% incidence of no-reflow [9]. Xie et al. evaluated 834 ACS patients and reported a no-reflow incidence ranging from 6.1% to 34%, depending on risk stratification, with the Canada ACS risk score significantly predicting no-reflow (AUC 0.75, *p* < 0.001) [10]. In a cohort of 3205 STEMI patients, the incidence of CNR reached 10.1% (*p* < 0.01) [11]. In Egypt, El Setiha et al. have reported a higher incidence of CNR reaching 20% in patients presented with STEMI (*p* = 0.02) [12]. The above discrepancy in the CNR incidence may be related to the difference in the definition or the modality of CNR diagnosis.

### 3.2. Pathophysiology

The development of CNR is centrally based on the emergence of the microvascular occlusion (MVO) with acute ischemic events [13]. Shortly, the coronary microcirculation, composed of arterioles, capillaries, and venules, is designed to regulate the myocardial perfusion during different stressors [14]. The development of MVO represents an interplay between endothelial injury, derangement of the endothelial barrier, and distal embolization together with reperfusion injury [15].

After cessation of the blood flow, the endothelial cells accumulate a wide spectrum of catabolic byproducts (e.g., lactate) within the intracellular and the interstitial space, forcing osmotic withdrawal of water from the intracapillary component to the intracellular and the interstitial fluid, creating a state of endothelial edema which leads to obstruction of the microvascular circulation [16]. Additionally, with more advanced ischemia, the sarcoplasmic reticulum begins to leak calcium into the cytoplasm, activating actin contraction [17]. The actin-mediated contraction forms multiple blebs over the swollen endothelial cells, resulting in loss of the pulsatile antegrade flow, further exacerbating the MVO [18]. In addition, actin contraction disrupts the endothelial barrier, exaggerating interstitial tissue edema [19].

In addition to endothelial barrier disruption, dissolution of the glycocalyx over the endothelial cells contributes to the development of the CNR. Glycocalyx is an important component of the endothelial barrier, representing a 0.5 µm thick carbohydrate-rich matrix that covers the endothelium surface throughout the capillary system [20]. Physiologically, the glycocalyx prevents the adhesion of circulating cells to endothelial cells. The highly hydrophilic nature of glycocalyx enables the creation of a relatively fixed (albeit exchangeable) water layer on the surface of endothelial cells, reducing the capillary hematocrit, facilitating the passage of blood through the capillaries [21]. During an ischemic event, the glycocalyx degrades secondary to reactive oxygen species and distal embolization, disturbing the endothelial barrier, facilitating migration of the inflammatory cells and platelets to the interstitial space [22].

Interestingly, growing evidence has highlighted the role of pericytes in the process of MVO. Pericyte is a contractile cell representing the second most frequent nonmyogenic cell found in the heart in vitro [23]. It plays an important role in the autoregulation of coronary microcirculation. Through its contractile function, pericytes play a pivotal role in CNR by constricting the coronary microcirculation even after the opening of the culprit vessels [24]. Targeting the pericyte is considered an attractive novel approach in managing patients with CNR [25]. Besides local cellular factors, the pooling of local and systemic vasoconstrictors impairs the microcirculation [26]. Multiple mechanisms have been proposed, including irreversible endothelial dysfunction, excessive adrenergic alpha receptors, and excessive vasoconstrictor substances (e.g., substance P) [18].

Moreover, during coronary interventions for ACS, the disintegration of the culprit thrombus paves the development of MVO by inducing distal embolization. Angiographically visible distal embolization is documented in 11% to 17% of primary percutaneous coronary intervention (PCI) procedures in patients with STEMI; however, its true incidence may be even higher [27,28]. The distal embolus is composed of platelet aggregates, erythrocytes, fibrin, cholesterol crystals, and inflammatory cells [29]. The distally embolized material ends up in the perfused myocardium, further exacerbating the degree of ischemia [30]. Distal embolization causes patchy microinfarcts that disproportionally impair the left ventricular function beyond the actual amount of damaged myocardium, increase infarct size, and are associated with poor clinical outcome [31].

**Figure 2:**
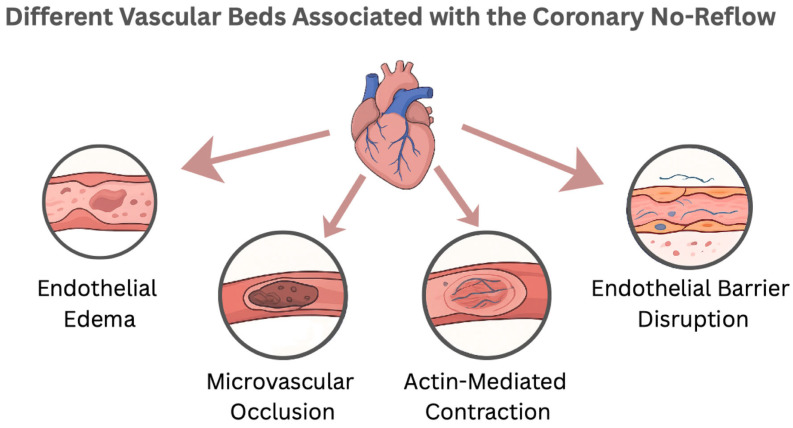
Vascular beds involved in the coronary no-reflow phenomenon, highlighting microvascular obstruction, endothelial dysfunction, and capillary damage following ischemia–reperfusion.

Additionally, the restoration of flow through the culprit artery is associated with increased risk of CNR through reperfusion-related injury. Interestingly, restoration of the blood flow to the deprived myocardial territory after sudden occlusion has a similar cellular effect to the interruption of the blood flow. Reperfusion injury is thought to occur secondary to oxidative stress. At the cellular level, reperfusion injury is associated with opening of the mitochondrial permeability transition pore, leading to loss of the mitochondrial inner membrane [32]. In addition, the CNR is closely linked to intramyocardial hemorrhage secondary to oxidative stress [33], correlated to an increase in vascular bed permeability secondary to disrupted gap junctions [34]. Intramyocardial hemorrhage is more common after prolonged severe ischemia followed by reperfusion, resulting in necrosis of endothelial cells, breakdown of the basal membrane, and destroyed microvessels [35]. In summary, the development of CNR represents an interplay with multiple, sometimes inevitable, pathophysiological pathways, enhancing microvascular occlusion and destroying the microcirculation basement membrane (Figure 2).

### 3.3. Risk Factors

#### Procedural-Related Risk Factors

A number of procedural variables have been found to predict the no-reflow phenomenon during PCI. Six pre-PCI variables were identified as independent predictors by the NORPACS risk score, which was developed from over 30,000 acute coronary syndrome PCI cases (Melbourne registry) and externally validated with over 440,000 cases from the BCIS registry. These variables included cardiogenic shock, delayed STEMI presentation (>195 min prior to PCI), vessel diameter < 2.5 mm, lesion location (left main or vein graft), and stent length ≥ 20 mm. Observed no-reflow rates in the development cohort and external validation cohort were roughly 22% and 27%, respectively, for high-risk patients (score ≥ 8) [36].

Thrombus grade ≥ 4, lesion length ≥ 35 mm, ischemic time ≥ 8 h, and reduced left ventricular ejection fraction (≤30%) were also found to be independent predictors of slow-flow/no-reflow (SF/NR) by the RK-SF/NR risk score, which was created in a multicenter cohort of 1711 STEMI patients. In this cohort, the incidence of SF/NR was roughly 28.8% [37].

### 3.4. Patient-Related Risk Factors

The no-reflow phenomenon has also been associated with patient-related factors. A Systemic Immune-Inflammation Index (SII) of 1036 was found to be an independent predictor of no-reflow in a study with 723 consecutive STEMI patients undergoing primary PCI (95% CI, 0.66–0.75, *p* < 0.001) [38]. Similarly, Gong et al. discovered that every 1 mg/L increase in plasma D-dimer was linked to 2.52 times higher odds of angiographic no-reflow in a group of 229 STEMI patients treated 2–7 days after symptom onset (OR 2.52; 95% CI: 1.16 to 5.47; *p* = 0.019). [39] A recent meta-analysis supports these results. It shows that higher D-dimer levels can predict no-reflow in STEMI patients receiving primary PCI. The prediction is significant but modest (95% CI: 1.001, 1.004; *p* < 0.05) [40].

### 3.5. Imaging Predictors and Advances

Certain plaque characteristics that are predictive of the no-reflow phenomenon during PCI have been found in recent studies using coronary computed tomography angiography (CTA). According to a study by Harigaya et al., no-reflow during PCI was independently predicted by low-attenuation plaque length greater than 4.7 mm on CTA (*p* < 0.001) [41]. These plaques are prone to distal embolization and are usually rich in lipids. In a different study, non-ruptured thin-cap fibroatheroma (TCFA) lesions were significantly linked to microvascular obstruction in comparison to non-TCFA plaques in 115 patients with acute coronary syndrome using optical coherence tomography (OCT) and cardiac magnetic resonance imaging (MRI) (43% vs. 9%, *p* = 0.012) [42].

More recently, clinical data have emerged confirming the predictive ability of intravascular ultrasound in the diagnosis of patients at increased risk of the no-reflow phenomenon when performing percutaneous coronary interventions. In this research, 707 patients with stable CAD, all of whom had 988 lesions, the combined ability of IVUS and coronary CTA to diagnose post-stent no-reflow was assessed [43].

The no-reflow phenomenon occurred in 22 lesions (2.2%) from 19 patients (2.7%). On IVUS, the presence of AP was characterized by low echogenicity without calcification and lacked a clear posterior border. AP on IVUS was considered to represent a lipid-rich, necrotic core plaque that is prone to distal embolization during PCI. The detection of IVUS-AP alone demonstrated a PPV of 15.7%, NPV of 99.8%, and overall diagnostic accuracy of 89.0% for predicting the occurrence of no-reflow. Similarly, CTA-derived v-LAP, as defined by a minimum density of <0 Hounsfield units, was associated with comparable diagnostic values (PPV 13.2%, NPV 99.6%, and accuracy 87.0%). Notably, the combination of findings according to IVUS AP and CTA v-LAP showed an improved predictive performance for no-reflow, with a PPV of 31.7%, NPV of 99.7%, and diagnostic accuracy of 95.5% [43].

These findings emphasize that both IVUS and CTA are complementary in determining the features of vulnerable plaques involved in the no-reflow phenomenon. While IVUS enables real-time, intravascular characterization of plaque morphology and composition, CTA provides a noninvasive assessment of low-attenuation, lipid-rich regions. The concordance between these modalities reinforces the concept that plaques with higher lipidic and necrotic content have a higher procedural risk. Therefore, this integrated imaging approach using IVUS and CTA may potentially improve the prediction of no-reflow events during PCI and lead to more personalized interventional strategies in patients with stable CAD [43].

The detection of MVO, an important sign of the no-reflow phenomenon after myocardial infarction (MI), has improved with recent advances in MRI technology. Native T1 mapping offers high diagnostic accuracy for identifying MVO after STMI. Carrick et al. found that native T1 values in the damaged heart muscle exceeding 1350 ms predicted MVO, with an area under the receiver operating characteristic curve (AUC) of 0.84 (95% CI: 0.78 to 0.90, *p* < 0.001). Elevated native T1, with a hazard ratio of 2.3 (95% CI: 1.4 to 3.7, *p* = 0.002), was also independently linked to negative changes in the left ventricle over six months and outperformed traditional LGE in predicting functional recovery. These findings suggest that native T1 mapping is a reliable, non-contrast marker for MVO and the extent of heart damage [44]. Furthermore, Masci et al. discovered that the interval between reperfusion and CMR caused a significant increase in T2 values in the infarct region. 163 patients with reperfused STEMI were the subjects of their study. Less than 43 h, 43–93 h, and > 93 h were the time tertiles, whose respective T2 values were 60.0 ± 4.9 ms, 63.5 ± 5.6 ms, and 64.8 ± 7.5 ms (*p* < 0.001). The same tertiles were also elevated in the unaffected myocardium, with values of 44.3 ± 2.8 ms, 46.1 ± 2.8 ms, and 46.1 ± 3.0 ms (*p* = 0.001). Across all tertiles, T2 values in the infarcted region were consistently significantly higher than those in the unaffected myocardium [45]. These findings confirm the diagnostic importance of native T2 mapping in cases of reperfusion injury and no-reflow risk stratification. The results show its ability to differentiate infarcted myocardium from healthy tissue and indicate how this changes over time.

Recent data point to the importance of comprehensive imaging in assessing microvascular dysfunction after PCI, and refractory no-reflow, in particular, is related to larger infarcts, poorer ventricular recovery, and higher mortality. A post hoc analysis of an RCT testing intracoronary epinephrine in refractory no-reflow provided unique insights into a non-invasive multimodal assessment. It combined dynamic SPECT to quantify functional flow parameters such as rest myocardial blood flow (RMBF), stress myocardial blood flow (SMBF), and global relative flow increase (gRFI) with cardiac MRI, characterizing structural injury, including infarct size, myocardial edema, and microvascular obstruction (MVO), beyond the traditional T1 and T2 mapping. Dynamic SPECT revealed globally impaired stress perfusion. Elevated LVEDP was strongly correlated with MRI-defined MVO (rs = 0.678, *p* = 0.001). Conventional metrics of flow reserve were insensitive; however, gRFI was significantly associated with MVO and thus represents a non-invasive marker of microvascular dysfunction. Collectively, these findings indicate the clinical benefit of combining functional imaging by SPECT with structural imaging by MRI in identifying microvascular injury after PCI [46].

## 4. Management

### 4.1. Pharmacologic Therapies

#### Antiplatelet Therapy

The fact that ticagrelor suppresses platelets more effectively than clopidogrel may influence the rate of NRF in STEMI patients during PPCI, according to the prior studies. A meta-analysis of 15 trials involving 4162 STEMI patients receiving PPCI assessed this. 2641 patients received a loading dose of clopidogrel (600 mg), whereas 1521 received ticagrelor (180 mg). Ticagrelor significantly reduced NRF compared to clopidogrel (95% CI: 0.15 to 0.39, *p* < 0.05), decreased post-procedural cTFC (95% CI: −8.89 to −6.91, *p* < 0.05), and improved final TIMI flow (95% CI: 1.40, 2.45, *p* < 0.05). Therefore, ticagrelor might outperform clopidogrel in improving coronary microvascular perfusion and preventing NRF during PPCI. Notably, bleeding episodes at 30 days (*p* = 0.82) and 180 days (*p* = 0.18) were not statistically different, indicating that ticagrelor’s increased antiplatelet activity did not increase the risk of bleeding [47].

### 4.2. Anticoagulation and the No-Reflow Phenomenon

When bivalirudin was used instead of unfractionated heparin (UFH) plus abciximab, there was no significant decrease in MVO in the HORIZONS-AMI cardiac magnetic resonance (CMR) substudy (28.6% vs. 34.8%, *p* = 0.63). Moreover, the two treatment groups showed similar infarct size, left ventricular systolic function, and remodeling indices at 7 days and 6 months after PCI. These findings suggest that reducing microvascular obstruction is unlikely to explain the clinical benefits of bivalirudin [48].

In contrast, a prospective study involving 42 STEMI patients undergoing primary PCI found that bivalirudin therapy (*n* = 21) was associated with a significant attenuation of myocardial edema volume in the acute phase when compared to UFH administration (*p* < 0.05). At one month following the intervention, myocardial edema was present in 33.3% of patients treated with UFH, whereas it was only 4.7% in the bivalirudin group (*p* < 0.05). By three months, the edema in the bivalirudin cohort had totally gone away, although two patients receiving UFH still had residual edema. Additionally, bivalirudin-treated patients had a significantly lower incidence and extent of MVO and intramyocardial hemorrhage (IMH) during the acute and follow-up periods (*p* < 0.05) [49].

The different results between the HORIZONS-AMI CMR substudy [48] and the EARLY-MYO-CMR trial [47] likely come from variations in study design, imaging methods, and sensitivity to microvascular damage. HORIZONS-AMI had a large overall size, but it included only 51 patients in its imaging substudy and found no significant difference in infarct size or microvascular obstruction with bivalirudin. In contrast, the smaller EARLY-MYO-CMR study had 42 participants and used T1/T2 mapping along with serial imaging. This study showed significantly reduced edema, microvascular obstruction, and intramyocardial hemorrhage with bivalirudin, all with *p*-values less than 0.05. These findings suggest that bivalirudin may reduce no-reflow through thrombin inhibition and microvascular protection. Earlier studies might have missed these effects because of limited imaging sensitivity and timing.

### 4.3. Inflammation Modulation

The relationship between inflammatory markers and the no-reflow phenomenon in STEMI patients receiving PCI was investigated in a recent meta-analysis. Higher levels of the platelet-to-lymphocyte ratio (PLR), leukocyte count, neutrophil count, red cell distribution width (RDW), and high-sensitivity C-reactive protein (Hs-CRP) were found to be strongly associated with an increased risk of no-reflow. Specifically, leukocytes, neutrophils, RDW, PLR, and Hs-CRP were all found to be independent predictors (*p* < 0.001). These findings suggest that microvascular blockage is significantly influenced by systemic inflammation. Patients who are more likely to experience no-reflow following primary PCI may be identified with the use of inflammatory biomarkers [50].

### 4.4. Mechanical and Device-Based Interventions

#### Aspiration Thrombectomy

**Figure 3:**
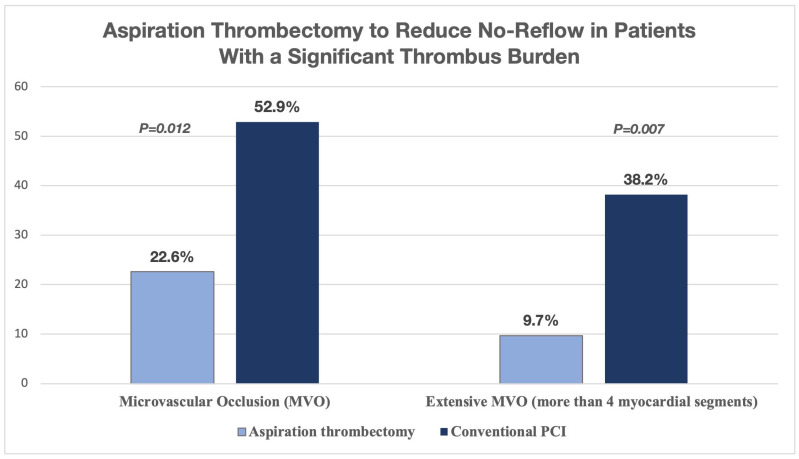
Aspiration thrombectomy to reduce no-reflow in patients with a significant thrombus burden [51].

In STEMI patients with a heavy thrombus burden, aspiration thrombectomy as an add-on treatment to primary PCI significantly lowered the occurrence and severity of MVO, which is a major factor in the no-reflow phenomenon. In this CMR-based study (n = 65), MVO was seen in 22.6% of patients who received aspiration compared to 52.9% in the conventional PCI group (*p* = 0.012). Furthermore, extensive MVO (more than 4 myocardial segments) was found in only 9.7% of patients undergoing aspiration thrombectomy, while it was 38.2% in the conventional group (*p* = 0.007) (Figure 3). These results support the targeted use of aspiration thrombectomy to reduce no-reflow in patients with a significant thrombus burden [51].

### 4.5. Distal Protection Devices (DPD)

The filter-type distal protection device Filtrap^®^ (Nipro Corporation, Osaka, Japan) was linked to lower rates of congestive heart failure (CHF) over a 2-year follow-up compared to patients treated without DP (*p* = 0.018) in a study of 164 patients undergoing PCI for acute myocardial infarction, even though there was no significant difference in post-procedural coronary flow. Using DP was found to be an independent predictor of lower CHF in a multivariate analysis (OR = 0.099; 95% CI, 0.02 to 0.42; *p* = 0.005). This may have a protective effect against microvascular damage caused by distal embolization. Distal protection may decrease the size of infarcts by preventing embolic debris from entering the perfused myocardial microvasculature, which would otherwise be at risk because emboli preferentially flow into vascularized tissue rather than already infarcted tissue. Major adverse cardiac events (MACE) did not differ significantly between groups, though (*p* = 0.238). These results suggest that, especially in high plaque-burden lesions, distal protection might reduce the effects of no-reflow-related sequelae like CHF [52]. Unfortunately, DPD are not used in contemporary primary PCI.

#### Emerging Device Technologies for Thrombus Management

The shortcomings of traditional aspiration and distal protection systems are being addressed by a number of innovative mechanical techniques, especially in situations where there is a high thrombotic burden or risk of distal embolization.

Focused ultrasound pulses are used in Histotripsy-Enhanced Aspiration to create controlled cavitation, which mechanically breaks up thrombotic material. Effective clot liquefaction has been shown in preclinical in vitro studies employing hollow cylindrical transducers, indicating increased aspiration efficiency and a possible decrease in embolic load during thrombectomy procedures [53].

Self-sensing aspiration Catheters, which use vacuum excitation for pressure sensing, have demonstrated a thrombus and catheter contact detection accuracy of 99.67% in benchtop models. In simulated clinical scenarios, this sensing method performed much better than traditional angiographic assessment, with an Odds Ratio of 2.86 and a *p*-value of 0.03. This technology may allow for real-time, guided thrombus removal while lowering the risk of distal embolization [54].

### 4.6. Stent and Balloon Technologies Impacting No-Reflow Incidence

Delaying stent implantation may reduce the risk of no-reflow and procedural complications in patients undergoing primary PCI or early invasive revascularization, according to emerging data. In a meta-analysis of 590 patients, delayed stenting significantly reduced procedure-related complications compared to immediate stenting (OR: 0.13; 95% credible interval [CrI]: 0.03, 0.36). During hospitalization, this approach did not increase the risk of major bleeding (OR: 0.81; 95% CrI: 0.01, 13.42) or major adverse cardiac events (OR: 0.40; 95% CrI: 0.09, 1.91) [55]. Delay in stenting was linked to a 45% relative decrease in myocardial infarction rates during hospitalization, with rates of 39% versus 60% in the single randomized trial (Relative Risk [RR]: 0.55; 95% CI: 0.39 to 0.80) [55]. This approach may reduce distal embolization and microvascular blockage, which are major factors in the no-reflow phenomenon. More large-scale randomized trials are needed to verify if this method leads to better long-term clinical outcomes.

The effectiveness and safety of giving intracoronary nicorandil with a perforated balloon (PB) were evaluated in a prospective observational study with 84 patients who developed CNR among 1789 patients undergoing PCI. The perforated balloon delivered 2–4 mg of nicorandil diluted in saline to the distal area over five minutes. Ten minutes after administration, researchers measured coronary flow quantitatively with the cTFC and qualitatively using the TIMI flow grade. Only 3.5% of cases showed no noticeable flow recovery. In contrast, 84.5% of cases successfully restored TIMI III flow, and 12% achieved TIMI II flow. cTFC decreased significantly from 52.9 ± 11 to 16.5 ± 5. Meanwhile, TIMI flow grades improved from a mean of 1.03 to 2.58 (both *p* < 0.001) [56].

**Figure 4:**
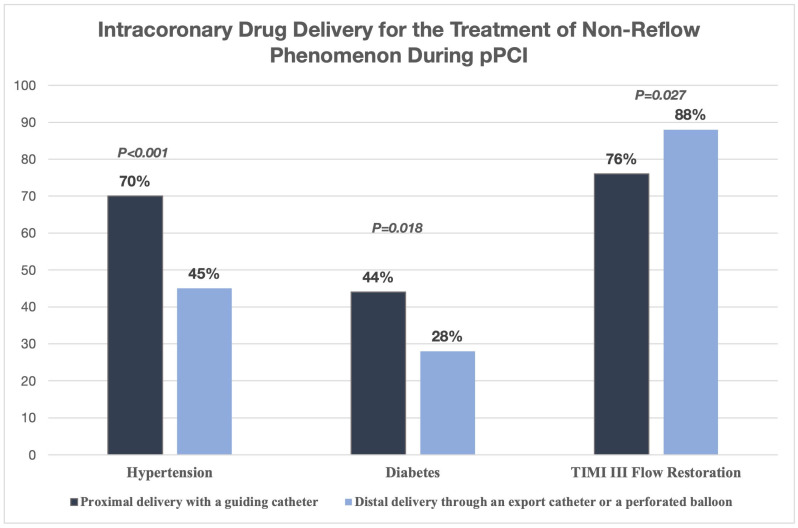
Intracoronary drug delivery for the treatment of non-reflow phenomenon during pPCI [37].

The possibility of delivering drugs directly into the coronary arteries can significantly influence treatment outcomes for slow flow and CNR during pPCI for STEMI. In a study with 200 patients who experienced NRF during primary PCI, researchers looked at the effectiveness of intracoronary drug delivery using two different techniques: proximal delivery through a guiding catheter and distal delivery through an export catheter or perforated balloon. The initial clinical and angiographic traits were similar between the two groups, though the proximal group had a higher percentage of hypertension (70% compared to 45%; *p* < 0.001) and diabetes (44% compared to 28%; *p* = 0.018). Despite these differences, the distal delivery method led to a significantly higher rate of TIMI III flow restoration (88% compared to 76%; *p* = 0.027) (Figure 4). These findings suggest that targeted distal drug delivery overcomes microvascular obstruction more effectively and restores optimal perfusion. This is likely because of improved local drug concentration at the site of microvascular dysfunction. Distal administration techniques, such as using a perforated balloon or a dedicated aspiration catheter, should be considered when possible in cases of intraprocedural NRF [37].

### 4.7. Intracoronary Epinephrine for Refractory No-Reflow During Primary PCI

Management of refractory coronary no-reflow is a major challenge during PPCI for STEMI. In a study involving 30 consecutive patients with severe refractory no-reflow (TIMI flow 0 to 1, myocardial blush grade 0 to 1), conventional treatments such as intracoronary nitrates, thrombectomy, glycoprotein IIb/IIIa inhibitors, and adenosine were ineffective. The use of intracoronary epinephrine showed better results in restoring coronary flow. Specifically, patients who received IC epinephrine achieved much higher rates of TIMI 2 to 3 flow (92.9%) than those who only received conventional therapy (31.3%; *p* = 0.004). Additionally, IC epinephrine led to a significant decrease in the 30-day composite endpoint of death or heart failure (35.7% vs. 81.25%). There were also significant improvements in left ventricular ejection fraction and ST-segment resolution (*p* = 0.01 for both). These results show that IC epinephrine may effectively reverse refractory no-reflow due to its strong vasodilating and inotropic effects. This could improve microvascular blood flow and help save heart muscle. Although this study is preliminary, it suggests that IC epinephrine needs more research as a potential treatment for no-reflow during PPCI, which could lead to better short- and long-term clinical outcomes [57].

### 4.8. Artificial Intelligence in Predicting and Managing No-Reflow After PCI

Recent studies have indicated that artificial intelligence (AI), especially machine learning and deep learning approaches, holds great promise for enhancing no-reflow diagnosis, management, and prediction. AI models are more accurate than conventional techniques at identifying high-risk patients by combining intricate clinical, procedural, and imaging data. For instance, algorithms that examine intravascular imaging data and pre-procedural variables can identify thrombus burden and microvascular obstruction, two major causes of no-reflow. In order to reduce the frequency and severity of no-reflow, these predictive capabilities allow for customized interventional strategies and prompt pharmacological interventions [56].

AI-driven tools improve intraprocedural decision-making and post-PCI prognostication in addition to risk prediction. Clinicians can optimize procedural techniques, like choosing distal protection devices or modifying medication regimens, with the help of real-time AI analysis of angiographic and perfusion imaging. AI models can also predict negative cardiovascular outcomes in patients who are not reflowing, which makes secondary prevention and individualized follow-up possible. Although encouraging, extensive clinical AI integration necessitates additional prospective validation and the creation of interpretable models to guarantee usability and trust. Finally, by offering precision-guided methods to reduce no-reflow and enhance patient outcomes, AI has the potential to completely transform PCI care [58].

### 4.9. Special Populations

No-reflow affects some high-risk groups more than others, especially the elderly, diabetic patients, and people with existing conditions like atrial fibrillation, hypertension, and heart failure. Older age is linked to a higher risk of no-reflow, probably because of an increased pro-inflammatory and pro-thrombotic state. This state is marked by higher levels of certain biomarkers, including fibrinogen, brain natriuretic peptide, leukocyte counts, and the neutrophil-to-lymphocyte ratio [59]. Diabetes mellitus also raises the chance of no-reflow, possibly due to microvascular dysfunction and damage to the endothelium caused by high blood sugar [60]. Furthermore, patients with atrial fibrillation are twice as likely to have no-reflow, demonstrating the relevance of systemic thrombo-inflammatory pathways [61]. Their risk is increased because these vulnerable people frequently have more severe coronary artery disease (CAD) and a higher thrombus burden. These characteristics indicate that, in order to improve reperfusion results and prevent adverse events in these patients undergoing primary PCI, specific prevention interventions and better medical care are required.

### 4.10. Prognostic Implications and Short- and Long-Term Outcomes

For STEMI patients, the no-reflow phenomenon following primary PCI is linked to a markedly higher long-term mortality rate. A study of 1406 STEMI patients found that 29% of them experienced no-reflow. In contrast to patients who had normal reperfusion, this was associated with a larger infarct size (15.0% vs. 8.0% of left ventricular volume, *p* < 0.001) [59]. In a 5-year follow-up, the no-reflow group experienced a significantly higher mortality rate (18.2%) compared to the reflow group (95% CI: 1.44–2.82; *p* < 0.001). Multivariate analysis, which accounted for infarct size and other factors, showed that no-reflow independently predicts 5-year mortality (95% CI: 1.17, 2.36; *p* = 0.004). These findings highlight that in STEMI patients after pPCI, no-reflow is an important prognostic marker that offers extra predictive value beyond infarct size [62].

In support of these results, a prospective study of 334 patients found that 11.1% experienced no-reflow, which was substantially associated with increased thrombogenicity as indicated by elevated platelet-fibrin clot strength (P-FCS ≥ 68 mm) on thromboelastography. Over a 3-year follow-up, patients with both no-reflow and high P-FCS had significantly worse long-term outcomes and a significantly higher risk of adverse clinical events (adjusted hazard ratio 6.65). These results highlight the pathophysiology of no-reflow and the detrimental effects of elevated thrombogenicity on cardiovascular events and long-term survival. In order to improve myocardial reperfusion and the long-term prognosis in this high-risk population, effective management of thrombogenicity may be a therapeutic target [63].

No-reflow occurred in 16.8% of 2463 patients in another study, which is consistent with earlier findings. Significantly, no-reflow was associated with a higher 30-day mortality rate (16.7%) than those without reflow (4.3%; *p* < 0.001). Higher Killip class, anterior myocardial infarction, longer symptom-to-door time, and older age were clinical predictors of no-reflow. These variables illustrate the relationship between ischemic damage and patient characteristics. Additionally, patients who presented with low systolic blood pressure were at greater risk. These findings demonstrate the grave consequences of no-reflow and the pressing need for focused strategies to lessen microvascular issues and increase short-term survival in this high-risk population [64].

### 4.11. Controversies and Unmet Needs

Current guidelines for managing no-reflow are inconsistent. Few clear strategies are recommended beyond the use of drugs that dilate blood vessels and mechanical support in certain cases. The 2020 ESC guidelines point out the shortage of strong evidence for many treatments. They stress the need for more large-scale trials to create standard procedures [65]. This situation highlights the challenges that interventionalists face when dealing with no-reflow. Often, their decisions depend more on their experience than on solid data.

The 2023 ESC Guidelines [66] on ACS recommend against routine prophylactic administration of pharmacologic agents, including glycoprotein IIb/IIIa inhibitors. Nonetheless, it is considered reasonable to use them as a bailout when there is no reflow (Class IIa, Level of Evidence C). Similarly, although there is little clinical evidence to support these recommendations, the 2018 EACTS Guidelines [1] on myocardial revascularization advocate selective thrombectomy and intracoronary vasodilators like adenosine, verapamil, and nitroprusside in certain clinical circumstances.

The 2024 ACC/AHA/SCAI Guidelines [67,68] for ACS, on the other hand, take a slightly more proactive stance: intracoronary or intravenous glycoprotein IIb/IIIa inhibitors might be justified in cases of slow flow or refractory no-reflow (Class IIa, Level C-LD), especially in thrombotic lesions. Evidence suggests that intracoronary epinephrine can restore TIMI grade 3 flow and enhance short-term clinical outcomes in cases of refractory no-reflow. In order to standardize no-reflow management, both sets of guidelines acknowledge the dearth of extensive randomized trials and urge more studies.

One major obstacle is the unpredictability of no-reflow. Microvascular spasm, endothelial dysfunction, reperfusion injury, and distal embolization are some of its intricate causes. It can be challenging to evaluate risk and choose a course of treatment because these variables can vary from patient to patient and lesion type. Additionally, the efficacy of current diagnostic tools is limited because they frequently detect no-reflow only after significant microvascular damage has occurred. Future research should examine the possibilities of novel biomarkers and imaging methods.

In conclusion, forecasting and managing no-reflow is still challenging. Guidelines are not generally accepted, treatment data is inconsistent, and the underlying causes are complicated. To improve outcomes for patients undergoing PCI, we need to address these issues with targeted research and innovative techniques.

### 4.12. Future Directions

Despite improvements in reperfusion methods, preventing and treating CNR and MVO are still major challenges in the current management of PCI patients. The reported incidence of CNR varies based on how sensitive the diagnostic tools are and when the evaluation takes place. While coronary angiography is the easiest method to access, it significantly underestimates the true prevalence of CNR. CMR is the most sensitive non-invasive tool. It allows for accurate measurement of infarct size, location, intramyocardial hemorrhage, and MVO. It is gaining recognition as a valuable tool for assessing prognosis and guiding treatment. Regular use of CMR in evaluating post-PCI STEMI particularly may help detect microvascular injury earlier and lead to more precise intervention strategies [63,69].

Future studies should focus on targeted management and prevention. It is crucial to change risk factors, especially those specific to each patient, such as high thrombotic burden, delayed presentation, diabetes, and chronic kidney disease. Researchers are exploring new medications that impact ischemia–reperfusion injury. These include calcium channel blockers, endothelin receptor antagonists, ATP-sensitive potassium channel openers like nicorandil, and recombinant insulin-like growth factor-1. Their goal is to reduce microvascular problems and cell damage. Additionally, intracoronary therapies are gaining popularity. For instance, the COAR trial [70] showed that intracoronary epinephrine could be a safe and effective alternative to adenosine for patients with hypotension who do not have reflow. This emphasizes the need for more comparative studies to improve agent selection and delivery methods.

To decrease distal embolization and preserve microcirculation, mechanical approaches such as distal embolic protection devices and microvascular flow enhancement techniques should be investigated. To develop targeted treatments, a deeper comprehension of the factors that contribute to no-reflow, such as inflammation, oxidative stress, endothelial dysfunction, and microembolization, is essential. To reduce the incidence and adverse long-term consequences of no-reflow in STEMI patients undergoing primary PCI, a combination of early risk assessment, enhanced imaging, medication therapy, and improved procedures will ultimately be essential.

Future plans will likely combine AI-driven risk assessment with new drug and regenerative treatments to effectively prevent and treat no-reflow, meeting the need for better microvascular outcomes in coronary procedures.

## 5. Conclusions

**Figure 5:**
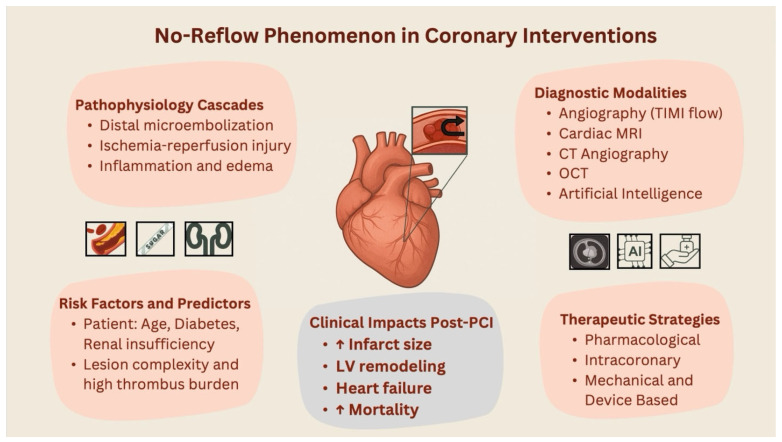
Central illustration: An overview of no-reflow during PCI that highlights important mechanisms, risk factors, diagnostic instruments, including cutting-edge AI techniques and treatment approaches. The black arrow indicates no-reflow; the small boxes on the right represent diagnostic imaging, AI, and pharmacological treatment; and the boxes on the left depict risk factors in the order of complex lesion, diabetes, and renal insufficiency.

The no-reflow phenomenon is a complex, multivariate, and poorly understood PCI companion across the ACS range. Its occurrence is consistently related with higher short- and long-term mortality, unfavorable ventricular remodeling, and bigger infarct size, especially in high-risk individuals with systemic inflammation, delayed presentation, or enhanced thrombotic burden (Figure 5). Despite advances in medication and procedural approaches, existing therapies provide patchy and ineffective protection against microvascular obstruction. Though detection is improved by diagnostic tools like cardiac MRI, predictive capabilities are still not optimal. While promising, emerging approaches like artificial intelligence-driven risk stratification, optimized intracoronary drug delivery, and mechanistically targeted therapies need strong validation. A multidisciplinary translational strategy will be necessary to address no-reflow in order to close ongoing gaps between pathophysiological understanding and successful clinical intervention.

## Abbreviations

ACS	Acute Coronary Syndrome
AI	Artificial Intelligence
CAD	Coronary Artery Disease
CHF	Congestive Heart Failure
CMR	Cardiac Magnetic Resonance
CNR	Coronary No-Reflow
cTFC	Corrected TIMI Frame Count
CTA	Computed Tomography Angiography
Hs-CRP	High-Sensitivity C-Reactive Protein
IC	Intracoronary
IMH	Intramyocardial Hemorrhage
MACE	Major Adverse Cardiac Events
MI	Myocardial Infarction
MRI	Magnetic Resonance Imaging
MVO	Microvascular Occlusion
OCT	Optical Coherence Tomography
PBT	Perforated Balloon Technique
PCI	Percutaneous Coronary Intervention
PLR	Platelet-to-Lymphocyte Ratio
RDW	Red Cell Distribution Width
STEMI	ST-Elevation Myocardial Infarction
TCFA	Thin-Cap Fibroatheroma
UFH	Unfractionated Heparin
